# CCDC68 Maintains Mitotic Checkpoint Activation by Promoting CDC20 Integration into the MCC

**DOI:** 10.1002/advs.202406009

**Published:** 2024-07-17

**Authors:** Qi Li, Qingzhou Chen, Tao Zheng, Fulin Wang, Junlin Teng, Haining Zhou, Jianguo Chen

**Affiliations:** ^1^ Key Laboratory of Cell Proliferation and Differentiation of the Ministry of Education College of Life Sciences Peking University Beijing 100871 China; ^2^ Key Laboratory of Epigenetic Regulation and Intervention Institute of Biophysics Chinese Academy of Sciences Beijing 100101 China; ^3^ Center for Quantitative Biology Peking University Beijing 100871 China

**Keywords:** CCDC68, CDC20, kinetochore, mitotic checkpoint complex, spindle assembly checkpoint

## Abstract

The spindle assembly checkpoint (SAC) ensures chromosome segregation fidelity by manipulating unattached kinetochore‐dependent assembly of the mitotic checkpoint complex (MCC). The MCC binds to and inhibits the anaphase promoting complex/cyclosome (APC/C) to postpone mitotic exit. However, the mechanism by which unattached kinetochores mediate MCC formation is not yet fully understood. Here, it is shown that CCDC68 is an outer kinetochore protein that preferentially localizes to unattached kinetochores. Furthermore, CCDC68 interacts with the SAC factor CDC20 to inhibit its autoubiquitination and MCC disassembly. Therefore, CCDC68 restrains APC/C activation to ensure a robust SAC and allow sufficient time for chromosome alignment, thus ensuring chromosomal stability. Hence, the study reveals that CCDC68 is required for CDC20‐dependent MCC stabilization to maintain mitotic checkpoint activation.

## Graphical abstract

The spindle assembly checkpoint (SAC) maintains chromosome segregation fidelity by manipulating unattached kinetochore‐dependent mitotic checkpoint complex (MCC) assembly. CCDC68, an outer kinetochore protein, localizes to unattached kinetochores and interacts with CDC20 to inhibit its autoubiquitination and MCC disassembly. This restrains anaphase‐promoting complex/cyclosome activation, ensuring a robust SAC for proper chromosome alignment and chromosomal stability.

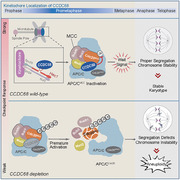

## 1 Introduction

The spindle assembly checkpoint (SAC) is the principal surveillance mechanism that puts anaphase on hold until all chromosomes are attached to spindle microtubules. Defects in this surveillance mechanism can result in chromosomal instability (CIN) and aneuploidy. The SAC surveys the state of kinetochore attachment to adjust anaphase promoting complex/cyclosome (APC/C) activation and prevent premature chromosome segregation.^[^ ^1^, ^2^ ^]^ The restraint of APC/C activity is executed by the mitotic checkpoint complex (MCC), which consists of MAD2, BUBR1, BUB3, and CDC20.^[^ ^3^, ^4^, ^5^, ^6^, ^7^ ^]^ The formation of the MCC is initiated by the recruitment of Monopolar Spindle 1 (MPS1) to unattached kinetochores,^[^ ^8^ ^]^ which subsequently phosphorylates MELT repeats in KNL1 to recruit BUB3–BUB1 and BUB3–BUBR1 in succession.^[^ ^9^ ^]^ The MAD1–MAD2 heterodimer binds to BUB1 to achieve the structural transformation of MAD2 from the open (O‐MAD2) to closed (C‐MAD2) form.^[^ ^10^ ^]^ Moreover, this conformational change in MAD2 requires its binding to CDC20^M^ (CDC20 functions in the MCC), which is the rate‐limiting step of MCC formation.^[^ ^11^ ^]^ Activation of the APC/C during mitosis relies on its association with the coactivator CDC20^A^ (CDC20 functions in the APC/C);^[^ ^12^ ^]^ therefore, when APC/C is suppressed by MCC. CDC20^A^ and CDC20^M^ are both bound to the APC/C.^[^ ^7^, ^13^ ^]^ Only the constant autoubiquitination of CDC20^M^ by APC/C^MCC^ can lead to MCC disassembly and allow the initiation of anaphase.^[^ ^14^, ^15^ ^]^

Once all kinetochores build the end‐on attachment with microtubules, the mitotic checkpoint is silenced, and APC/C‐CDC20^A^ is reactivated to promote the ubiquitination and degradation of securin and cyclin B1;^[^ ^16^, ^17^ ^]^ this in turn initiates anaphase onset. As the checkpoint is silenced, cessation of MCC production coincides with the turnover of the APC/C‐bound MCC.^[^ ^1^ ^]^ The disruption of MCC production relies on bioriented kinetochores, which prevent MPS1 recruitment and increase PP1/PP2A phosphatase activity.^[^ ^5^ ^]^ The turnover of existing MCCs occurs via two parallel pathways:^[^ ^18^ ^]^ one pathway involves APC15 driving the autoubiquitination of CDC20 to promote the disassembly of the APC/C‐bound MCC;^[^ ^14^, ^19^ ^]^ the other pathway is mediated by p31^comet^/TRIP13, which intercepts and disassembles free MCC.^[^ ^20^ ^]^ This process prevents the MCC from interacting with the APC/C by changing the conformation of C‐MAD2 back to O‐MAD2, which in turn disrupts the association between CDC20 and MAD2.^[^ ^20^ ^]^ The “pool” of the MCC on an unattached kinetochore is sufficient to antagonize its rapid turnover, whereas the mechanism remains unclear.

Coiled‐coil domain‐containing 68 (CCDC68, also known as se57‐1) was first identified as a tumor‐associated antigen in cutaneous T‐cell lymphomas.^[^ ^21^ ^]^ Our previous work showed that CCDC68 localizes to centriole subdistal appendages and functions in microtubule anchoring during interphase.^[^ ^22^ ^]^ In the present study, we demonstrate that CCDC68 is recruited to unaligned kinetochores during prometaphase. CCDC68 interacts with CDC20 and MAD2 to inhibit the autoubiquitination of CDC20 and restrains the turnover of the MCC on APC/C, thus maintaining the strength of the SAC and the fidelity of chromosome alignment. These findings demonstrate that CCDC68 acts as a suppressor of MCC disassembly, thus ensuring the accurate transmission of genetic material.

## 2 Results

### 2.1 CCDC68 is Localized at the Outer Plate of Unaligned Kinetochores

**Figure 1:**
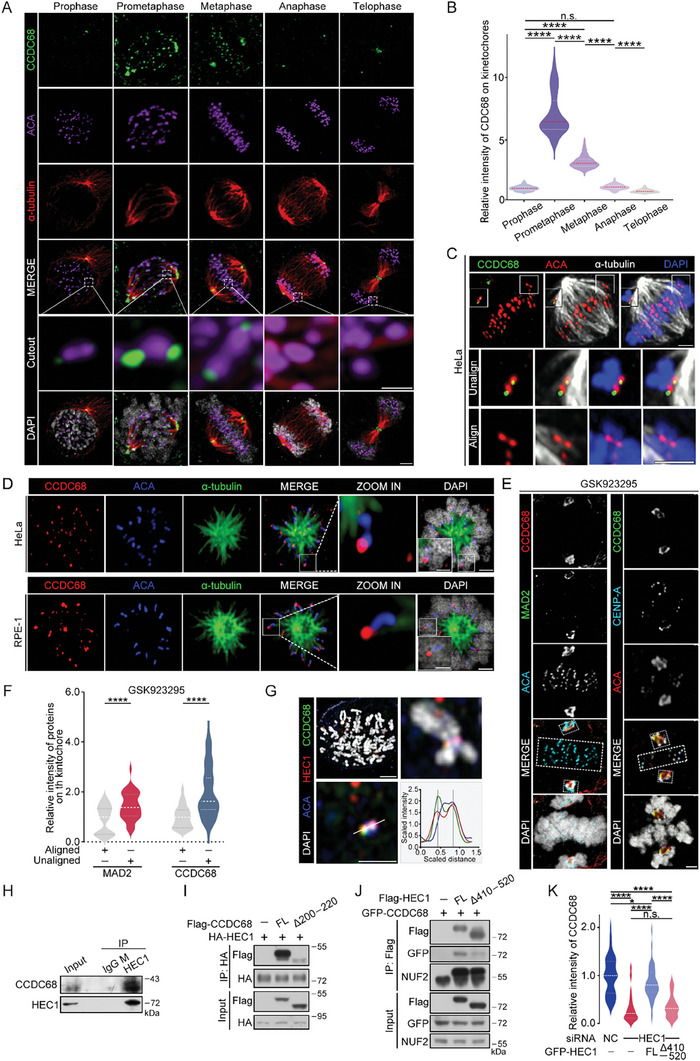
CCDC68 localizes to the outer plate of unaligned kinetochores by interacting with HEC1. A) Immunofluorescence staining for CCDC68 (green), ACA (purple), and α‐tubulin (red) in wild‐type (WT) HeLa cells at different stages of mitosis. DNA was stained with DAPI (white). Scale bars, 2 µm, scale bars in the cut‐out, 0.5 µm. B) Quantification of the fluorescence intensity of CCDC68 staining on kinetochores shown in (A). More than 100 kinetochores from 10 cells were analyzed. C) Immunofluorescence staining for CCDC68 (green), ACA (red), and α‐tubulin (white) in WT HeLa cells. DNA was stained with DAPI (blue). Scale bars in the full images, 2 µm; scale bars in the cut‐out, 0.5 µm. D) Immunofluorescence staining for CCDC68 (red), α‐tubulin (green), and ACA (blue) in monastrol‐treated HeLa or RPE‐1 cells. DNA was stained with DAPI (white). Scale bar in the full image, 2 µm; Scale bar in the cut‐out, 0.5 µm. E) Immunofluorescence staining for CCDC68 (green), CENP‐A (cyan), and ACA (red) in HeLa cells treated with GSK923295 for 3 h (right panel); immunofluorescence staining for CCDC68 (red), MAD2 (green), and ACA (cyan) in HeLa cells treated with GSK923295 for 3 h (left panel). DNA was stained with DAPI (white). The aligned kinetochore is included in dotted boxes at the equator plate. Unaligned kinetochore is included in dotted boxes at the spindle poles. Scale bars, 2 µm. F) Quantification of the relative fluorescence intensity of CCDC68 and MAD2 staining on the kinetochores shown in (E). More than 100 kinetochores from 10 cells were analyzed. G) Immunofluorescence staining for CCDC68 (green), HEC1 (red), and ACA (blue) in HeLa cells after the chromosome spread assay. DNA was stained with DAPI (white). The quantitative plot shows the fluorescence intensity along the white line of the image. Scale bar in the full image, 2 µm; Scale bar in the cut‐out, 0.5 µm. H) Lysates from HeLa cells were subjected to immunoprecipitation (IP) with an anti‐IgG (mouse) or anti‐HEC1 antibody, and the resulting samples were analyzed by immunoblotting with the indicated antibodies. I) Lysates from HEK293T cells cotransfected with Flag‐vector, Flag‐CCDC68‐Full length (FL), or Flag‐CCDC68‐Δ200–220 and HA‐HEC1 were subjected to coimmunoprecipitation, and the resulting samples were analyzed by immunoblotting with the indicated antibodies. J) Lysates from HEK293T cells cotransfected with Flag‐vector, Flag‐HEC1‐Full length (FL), or Flag‐ HEC1‐Δ410–520 and GFP‐CCDC68 were subjected to coimmunoprecipitation, and the resulting samples were analyzed by immunoblotting with the indicated antibodies. K) Quantification of the fluorescence intensity of CCDC68 staining on kinetochores in (Figure S2L, Supporting Information). More than 100 kinetochores from ten cells were analyzed. All the data are presented as the means of the indicated biological replicates; error bars represent the means ± SEMs. Statistical analyses were performed using Student's *t*‐test for (F) and using one‐way ANOVA for (B) and (K). n.s., not significant. *p < 0.05, **p < 0.01, ***p < 0.001, ****p < 0.0001.

CCDC68 is highly conserved across multiple species ranging from mice to humans (Figure S1A, Supporting Information). We noticed that CCDC68 predominantly localized to kinetochores during mitosis as shown by its colocalization with ACA, which acts as kinetochore marker (**Figure** **1**A,B). Moreover, CCDC68 was periodically localized to kinetochores, and the colocalization intensity increased from prophase to prometaphase and decreased after that (Figure 1A,B). However, the protein level of CCDC68 was relatively constant throughout the cell cycle (Figure S1B, Supporting Information). Ectopic mEmerald‐CCDC68 and Flag‐CCDC68 were also prominently localized to kinetochores during prometaphase (Figure S1C, Supporting Information). We then monitored the dynamics of mEmerald‐CCDC68 in live HeLa cells by high‐speed spinning‐disk confocal microscopy, and the results further showed CCDC68 enrichment at the kinetochores after nuclear envelope breakdown, with its intensity gradually decreasing as mitosis progressed from prometaphase to metaphase (Figure S1D, Supporting Information). Following the alignment of all chromosomes at the spindle equator, CCDC68 was barely detectable on kinetochores (Figure S1D, Supporting Information).

Further immunofluorescence experiments showed that only the unaligned kinetochores exhibited CCDC68 localization rather than the kinetochores aligning at the equatorial plate (Figure 1C). CCDC68 was only present on the side of ACA without an α‐tubulin signal but not on the other side of ACA that was in contact with α‐tubulin in monopolar HeLa and RPE‐1 cells after monastrol treatment (Figure 1D), suggesting that CCDC68 was preferentially enriched at unattached or lateral kinetochores rather than at mature end‐on kinetochores. A similar localization preference was observed in HCT116 cells (Figure S1E, Supporting Information).

Next, we added GSK923295, an inhibitor of the motor protein CENP‐E,^[^ ^23^ ^]^ to induce syntelic kinetochore formation. Most of the chromosomes were aligned to the equatorial plates and displayed no CCDC68 signal (Figure 1E,F). CCDC68 was specifically localized to the few unaligned kinetochores (Figure 1E,F). In addition, immunofluorescence showed that when localized to unaligned kinetochores, CCDC68 aggregated around spindle poles and did not overlap with centrosomes (Figure S1F, Supporting Information); this localization pattern was consistent with that of MAD2/MAD1 (Figure 1E,F; Figure S1G, Supporting Information), which are checkpoint factors that disassociate from kinetochores with end‐on attachment,^[^ ^24^ ^]^ further demonstrating that CCDC68 preferentially localizes to unaligned kinetochores.

Kinetochores are composed of three distinct layers: the inner plate, outer plate, and fibrous corona.^[^ ^25^ ^]^ To specify the exact location of CCDC68, we performed coimmunostaining for CCDC68 with the inner plate marker ACA,^[^ ^26^ ^]^ the fibrous corona protein ZW10,^[^ ^27^ ^]^ and the outer plate proteins BUBR1,^[^ ^4^ ^]^ MAD1,^[^ ^5^ ^]^ and HEC1.^[^ ^28^ ^]^ CCDC68 localized to the outside of ACA and the inside of ZW10 (Figure S1H, Supporting Information). CCDC68 colocalized substantially with HEC1 (Figure 1G; Figure S1H, Supporting Information) and localized more closely to the interior of the kinetochore than did BUBR1 and MAD1 (Figure S1H, Supporting Information).

CCDC68 and HEC1 were copurified, as shown by coimmunoprecipitation assays (Figure 1H; Figures S2A and S2B, Supporting Information). To determine the regions of CCDC68 that are required for its association with HEC1, a series of CCDC68 truncation mutants were tested (Figure S2C, Supporting Information). The 200–220 residues in the C‐terminus of CCDC68 were found to be responsible for its binding to HEC1 (Figure 1I; Figure S2D, Supporting Information). Furthermore, CCDC68 associated with the C‐terminal residues 410–642 of HEC1 (Figure S2E,F, Supporting Information), which compose a substantial portion of the coiled‐coil domain.^[^ ^29^ ^]^ Further mapping assays revealed that region 410–520 was responsible for the interaction between HEC1 and CCDC68 (Figure 1J; Figure S2G, Supporting Information). Consistently, knockdown of HEC1 resulted in reduced localization of CCDC68 to kinetochores during prometaphase (Figure S2H,J, Supporting Information), while knockdown of CCDC68 did not affect the localization of HEC1 to kinetochores (Figure S2I,K, Supporting Information). We further generated HEC1‐knockdown HeLa cells that stably expressed siRNA‐resistant full‐length HEC1 or the HEC1‐Δ410–520 mutant. As expected, compared with full‐length HEC1, the HEC1‐Δ410–520 mutant failed to rescue CCDC68 localization to kinetochores (Figure 1K; Figure S2L, Supporting Information), suggesting that CCDC68 is recruited to the outer kinetochore by HEC1.

### 2.2 CCDC68 Prevents Immature Anaphase Onset and Chromosome Lagging

**Figure 2:**
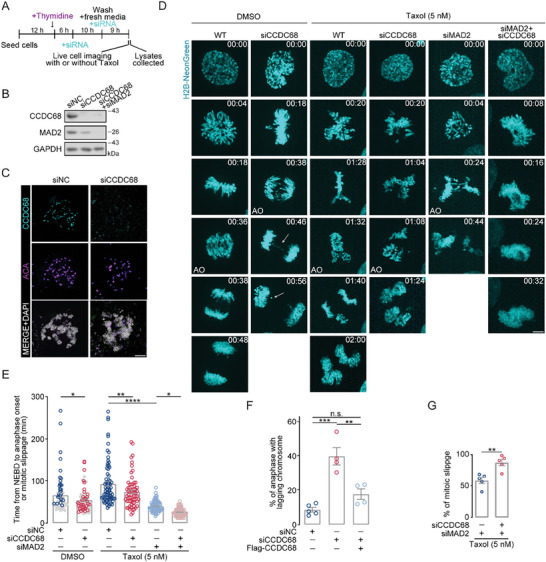
CCDC68 depletion accelerates anaphase onset and leads to chromosome lagging. A) Schematic diagram of the compound and siRNA treatment process in (B) and (D). B) Immunoblots showing the depletion of CCDC68 (siCCDC68) alone or CCDC68 and MAD2 (siCCDC68+siMAD2) in HeLa cells. GAPDH served as the loading control. NC, negative control. C) Immunofluorescence staining for CCDC68 (cyan) and ACA (purple) in HeLa cells transfected with siNC or siCCDC68. Scale bars, 2 µm. D) Live‐cell imaging of wild‐type (WT) HeLa cells or HeLa cells transfected with siNC, siCCDC68, siMAD2, or both siCCDC68 and siMAD2 and then subjected to drug treatment. Cells also stably expressed H2B‐NeonGreen. The time of nuclear envelope breakdown was set to zero; the time is presented in minutes on each channel image. The white arrow indicates mitotic defects. AO: anaphase onset. Scale bars, 5 µm. E) The time from NEBD to anaphase onset or mitotic slippage was quantitated from the time‐lapse analysis in (D). The gray circles represent the normal mitotic cells, and the blue circles and red circles represent the cells with mitotic defects in DMSO treatment groups. F) Quantification of the percentage of mitotic slippage from the dataset as in (D) and (Figure S3D, Supporting Information). G) Quantification of the percentage of anaphase with lagging chromosomes from the dataset as in (D). All the data are presented as the means of the indicated biological replicates; error bars represent the means ± SEMs. Statistical analyses were performed using Student's *t*‐test for (G) and using one‐way ANOVA for (E) and (F). n.s., not significant (*p* > 0.05), *p < 0.05, **p < 0.01, ***p < 0.001, ****p < 0.0001.

To analyze the functions of CCDC68 in mitosis, we conducted RNAi assay to deplete CCDC68 in HeLa cells stably expressing NeonGreen‐tagged histone H2B (**Figures** **2**A–C; Figures S3A–C, Supporting Information). Cells were synchronized to the G1/S phase by thymidine block, and the block was then released in the presence of taxol to induce M‐phase arrest (Figure 2A). Live‐cell imaging revealed a shortened mitotic duration in the CCDC68‐knockdown cells compared to the control cells (Figure 2D,E). The percentage of cells in anaphase with lagging chromosomes increased to ∼40% after CCDC68 depletion compared to ≈10% in wild‐type cells, and this effect could be rescued by Flag‐CCDC68 expression (Figure 2F; Figure S3D, Supporting Information). Following the enforced activation of SAC by treatment with 5 nM taxol,^[^ ^30^ ^]^ the time from nuclear envelope breakdown to anaphase onset was increased to ≈93 min in control cells (Figure 2D,E), and most cells did not initiate anaphase until all the chromosomes were congressed. However, knockdown of CCDC68 shortened this time frame to ≈73 min and resulted in incomplete chromosome congression at the time of anaphase onset (Figure 2D,E). Given that the localization pattern of CCDC68 to unattached kinetochores is consistent with that of MAD2 (Figure 1E), we further investigated whether CCDC68 cooperated with MAD2 to regulate mitotic progression. We observed that the percentage of cells with mitotic slippage, which involves cells with deficient SAC slipping out of mitosis rapidly in the absence of proper chromosome segregation and cytokinesis,^[^ ^31^, ^32^ ^]^ reached nearly 100% after depletion of both CCDC68 and MAD2, while depletion of MAD2 caused mitotic slippage in only ≈50% of cells (Figure 2G). These results suggested that the impairment of SAC signaling caused by CCDC68 depletion is not entirely dependent on the role of MAD2 in retarding SAC silencing. Taken together, these data suggest that depletion of CCDC68 attenuates SAC strength and disrupts accurate chromosome alignment.

### 2.3 CCDC68 Interacts with CDC20 to Maintain MCC Integrity

**Figure 3:**
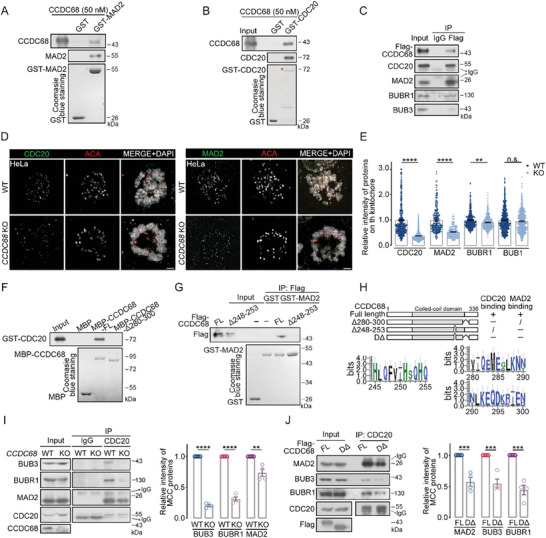
CCDC68 stabilizes MCC by interacting with CDC20 and MAD2. A) Purified CCDC68 (50 nM) was incubated with either GST or GST‐MAD2. The samples were analyzed by Coomassie blue staining and immunoblotting with the indicated antibodies. B) Purified CCDC68 (50 nm) was incubated with either GST or GST‐CDC20. The samples were analyzed by Coomassie blue staining and immunoblotting with the indicated antibodies. The red asterisk indicates GST‐CDC20. C) Lysates from CCDC68‐knockout (KO) cells stably expressing Flag‐CCDC68 were synchronized to prometaphase and subjected to immunoprecipitation (IP) with an anti‐Flag antibody, and samples were analyzed by immunoblotting with the indicated antibodies. D) Immunofluorescence images of wild‐type (WT) and CCDC68‐KO HeLa cells stained for CDC20 (green, left panel) or MAD2 (green, right panel) and ACA (red). DNA was stained with DAPI (blue). Scale bars: 2 µm. E) Quantification of the relative fluorescence intensity of proteins on kinetochores shown in (D), (Figure S4F, Supporting Information), and (Figure S4G, Supporting Information). More than 100 kinetochores from ten cells were analyzed. F) Purified MBP, MBP‐CCDC68‐Full length (FL) or MBP‐CCDC68‐Δ280–300 was incubated with GST‐CDC20. The samples were analyzed by Coomassie blue staining and immunoblotting with the indicated antibodies. G) Lysates of HEK293T cells expressing Flag‐CCDC68‐Full length (FL) or Flag‐CCDC68‐Δ248–253 were incubated with purified GST or GST‐MAD2. The samples were analyzed using immunoblotting with the indicated antibodies and Coomassie blue staining. H) Alignment of residues 245–255 and 280–300 from model organisms. The size of the amino acid letters represents the level of conservation. I) Lysates from WT and CCDC68‐KO HeLa cells were synchronized to prometaphase and subjected to IP with an anti‐CDC20 antibody, and the resulting samples were analyzed by immunoblotting with the indicated antibodies. Quantification of the relative intensity of MCC proteins is shown. J) Lysates from CCDC68‐KO cells stably expressing Flag‐CCDC68‐Full length (FL) or Flag‐CCDC68‐DΔ (Δ248–253 and Δ280–300) were synchronized to prometaphase and subjected to IP with an anti‐CDC20 antibody, and the protein levels in the resulting samples were measured by immunoblotting. Quantification of the relative intensity of MCC proteins is shown. All the data are presented as the means of the indicated biological replicates; error bars represent the means ± SEMs. Statistical analyses were performed using Student's *t*‐test for (E), (I), and (J). n.s., not significant. *p < 0.05, **p < 0.01, ***p < 0.001, ****p < 0.0001.

We next investigated the effect of CCDC68 on the SAC. As the SAC is activated by unaligned kinetochores through the constant production of MCCs (which contain CDC20, MAD2, BUB3, and BUBR1), we first tested whether CCDC68 is associated with the MCC; in vitro pull‐down assays showed that CCDC68 interacts with MAD2 and CDC20 (**Figure** **3**A,B). We further generated CCDC68‐knockout HeLa cells stably expressing Flag‐CCDC68 (Figure S4A–E, Supporting Information), and performed coimmunoprecipitation. We found that CCDC68 strongly interacted with CDC20 and MAD2 and weakly interacted with BUB3 and BUBR1 (Figure 3C). In addition, loss of CCDC68 reduced the kinetochore localization of MCC components, including CDC20, MAD2, and BUBR1, during prometaphase (Figure 3D,E; Figure S4F, Supporting Information) but barely affected the recruitment of BUB1, MIS12,^[^ ^33^ ^]^ or the inner plate protein CENP‐C (Figure 3E; Figure S4G–J, Supporting Information); however, knockdown of CDC20 or MAD2 did not affect the localization of CCDC68 to kinetochores (Figure S5A–F, Supporting Information), suggesting that CCDC68 facilitates the accumulation of MCC components at kinetochores.

We next sought to determine the region of CCDC68 that binds to CDC20 and MAD2 by testing a series of truncation mutants. The regions encompassing residues 280–300 and 248–253 (ABBA‐like motif) of CCDC68, which represent two evolutionarily conserved consensus sequences, were responsible for its binding to CDC20 and MAD2, respectively (Figure 3F–H; Figure S5G–L, Supporting Information). The IR motif of CDC20 binds to the APC/C and is required for CDC20 autoubiquitination and MCC stabilization.^[^ ^34^, ^35^ ^]^ The point mutant of CDC20 (I498A/R499A, IRAA) abrogated its association with CCDC68 (Figure S5M,N, Supporting Information), suggesting that the IR motif of CDC20 is responsible for its interaction with CCDC68.

To investigate the role of CCDC68 in the MCC, we tested the integrity of the MCC after *CCDC68* knockout. Depletion of CCDC68 disrupted the interaction between CDC20 and BUBR1, BUB3, or MAD2 in mitotic HeLa cells, as shown by immunoprecipitation (Figure 3I). Furthermore, in CCDC68‐knockout HeLa cells stably expressing Flag‐CCDC68‐DΔ (Δ280–300 and Δ248–253), which was unable to bind to CDC20 and MAD2 (Figure 3F–H), the association of CDC20 with MAD2, BUBR1, and BUB3 was also diminished (Figure 3J). Moreover, the expression of CCDC68‐Δ200–220, which lacks the ability to interact with HEC1 (Figure 1I), did not restore the integrity of MCC in CCDC68‐knockout cells (Figure S5O, Supporting Information). These data suggest that the interaction of CCDC68 with CDC20 and MAD2 is required for MCC integrity.

### 2.4 CCDC68 Contributes to MCC‐Dependent Catalytic Restriction of the APC/C

**Figure 4:**
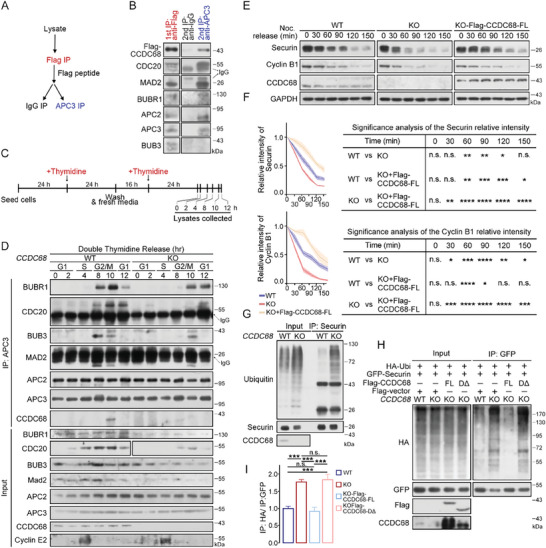
CCDC68 limits APC/C‐dependent ubiquitinated degradation by inhibiting MCC disassembly. A) Schematic diagram of the sequential immunoprecipitation (IP) process. B) Lysates from HeLa cells were subjected to IP with an anti‐Flag antibody and eluted with 50 µm Flag‐peptide. The eluent was incubated with Protein G beads coated with an IgG or APC3 antibody. The samples were analyzed by immunoblotting with the indicated antibodies. C) Schematic diagram of the compound treatment process in (D). D) Wild‐type (WT) and CCDC68‐knockout (KO) HeLa cells were synchronized by double‐thymidine treatment. The cells were then harvested, lysates were subjected to IP with an anti‐APC3 antibody, and the protein levels in the resulting samples were measured by immunoblotting. E) WT, CCDC68‐KO, and CCDC68‐KO HeLa cells stably expressing Flag‐CCDC68‐Full Length (FL) were synchronized by thymidine‐nocodazole treatment. Mitotic shake‐off cells were collected, washed twice with PBS, and released in fresh medium. The cells were then harvested at various time points as indicated. The cells were then processed, and the protein levels in the resulting samples were measured by immunoblotting. GAPDH served as the loading control. F) Quantification of the relative intensity of securin and cyclin B1 shown in (E). G) Lysates from WT and CCDC68‐KO HeLa cells were subjected to IP with an anti‐securin antibody, and the samples were analyzed using immunoblotting with the indicated antibodies. H) WT, CCDC68‐KO, and CCDC68‐KO cells stably expressing Flag‐CCDC68‐Full length (FL) or Flag‐CCDC68‐DΔ were transfected with HA‐ubiquitin (HA‐Ubi) and GFP‐securin. The cells were subjected to IP with an anti‐GFP antibody. The level of exogenous GFP‐securin ubiquitin was detected with an anti‐HA antibody. I) Quantification of the relative intensity shown in (H). All the data are presented as the means of the indicated biological replicates; error bars represent the means ± SEMs. Statistical analyses were performed using one‐way ANOVA for (F) and (I). n.s., not significant, *p < 0.05, **p < 0.01, ***p < 0.001, ****p < 0.0001.

Since APC/C activation is restored via its disassociation from the MCC, we examined whether CCDC68 regulates the interaction status between the APC/C and MCC complexes. With anti‐Flag‐CCDC68 immunoprecipitation samples, we performed a second immunoprecipitation with an anti‐APC3 antibody, and the results showed that Flag‐CCDC68 was copurified with the APC/C as well as the subunits of the MCC, including CDC20, BUBR1, and MAD2 (**Figure** **4**A,B), suggesting that CCDC68, MCC, and APC/C can form a mega‐complex in mitotic cells. The interactions of APC3 with BUBR1, CDC20, BUB3, and MAD2 were significantly reduced in CCDC68‐knockout cells during the G2/M phase (Figure 4C,D), but the interactions of APC3 with APC2, APC4, APC5, and APC8 were similar to those in wild‐type cells (Figures 4C,D; Figure S6, Supporting Information), suggesting that the loss of CCDC68 compromises the binding between the APC/C and MCC without affecting the integrity of the APC/C. As expected, the interaction between CCDC68 and the APC/C‐MCC was observed only during the G2/M phase (Figure 4D).

As an E3 ligase, APC/C can ubiquitinate and degrade cyclin B1 and securin;^[^ ^16^, ^17^ ^]^ these proteins were rapidly degraded in CCDC68‐knockout cells, and could be rescued by CCDC68 expression (Figure 4E,F). Consistently, the ubiquitination of endogenous securin was significantly increased in CCDC68‐knockout cells, which could be rescued by CCDC68‐Full length expression, but not by CCDC68‐DΔ (Figure 4G–I), suggesting that CCDC68 inhibits the ubiquitination activity of the APC/C, which requires the interaction of CCDC68 with the MCC. Taken together, CCDC68 restrains APC/C‐dependent degradation of substrates by limiting MCC disassembly.

### 2.5 CCDC68 Stabilizes the MCC by Blocking Ubiquitination‐Dependent CDC20 Turnover

**Figure 5:**
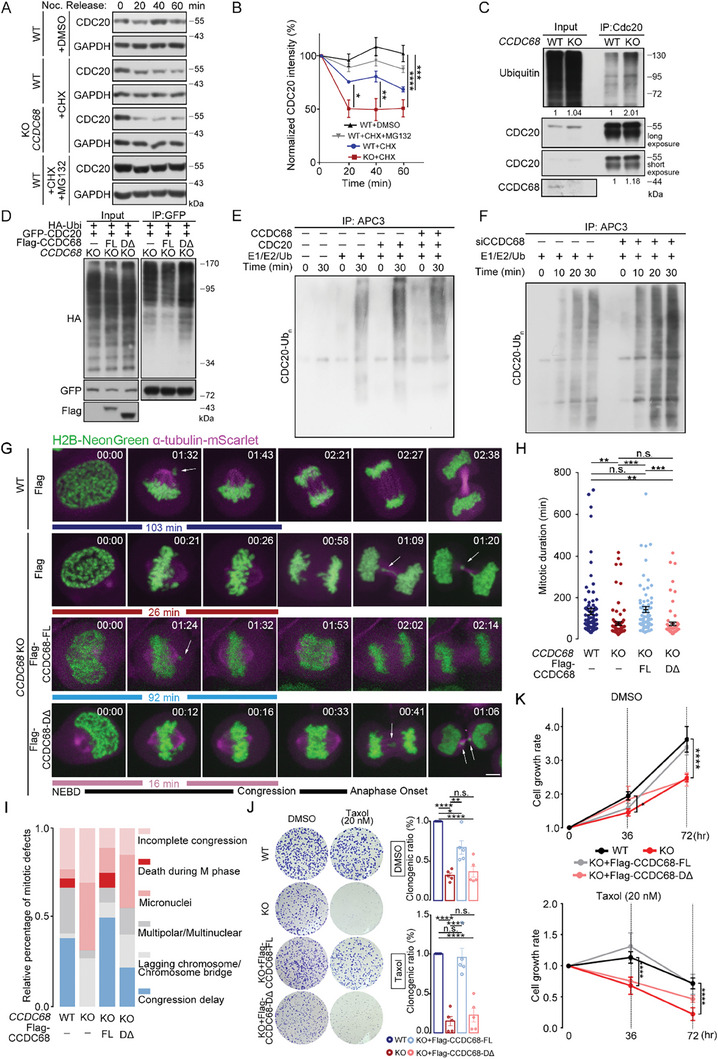
CCDC68 stabilizes the MCC by inhibiting the ubiquitination‐dependent turnover of CDC20. A) Cells were treated with cycloheximide (CHX), as indicated, to block protein synthesis. The proteasome inhibitor MG132 was added as indicated. The immunoblot shows CDC20 degradation in wild‐type (WT) and CCDC68‐KO cells that were arrested in prometaphase. B) Quantification of the relative intensity is shown in (A). C) Lysates from WT and CCDC68‐KO HeLa cells were subjected to IP with an anti‐CDC20 antibody, and the samples were analyzed by immunoblotting with the indicated antibodies. D) CCDC68‐KO cells stably expressing Flag‐vector, Flag‐CCDC68‐Full length (FL) or Flag‐CCDC68‐DΔ (Δ248–253 and Δ280–300) were cotransfected with HA‐ubiquitin (HA‐Ubi) and GFP‐CDC20. Lysates were subjected to IP with an anti‐GFP antibody, and samples were analyzed by immunoblotting with the indicated antibodies. E) The APC/C was immunoprecipitated by anti‐APC3 antibody‐incubated beads from the extracts of cells that were synchronized by double‐thymidine treatment. The APC/C was supplemented with purified CCDC68, CDC20, or E1/E2/Ub and incubated for 0 or 30 min. After incubation, the samples were analyzed by immunoblotting with the indicated antibodies. CDC20‐Ub_n_, ubiquitinated CDC20. F) WT or CCDC68‐depleted HeLa cells were synchronized by double‐thymidine treatment to collect mitotic cells. The APC/C was immunoprecipitated with anti‐APC3 antibody‐incubated beads and supplemented with E1/E2/Ub, and then incubated for the indicated time points. After incubation, the samples were analyzed by immunoblotting with the indicated antibodies. CDC20‐Ub_n_, ubiquitinated CDC20. G) Live‐cell imaging of wild‐type (WT), CCDC68‐knockout (KO), and CCDC68‐KO HeLa cells stably expressing Flag‐CCDC68‐Full length (FL) or Flag‐CCDC68‐DΔ. The cells also stably expressed H2B‐NeonGreen and α‐tubulin‐mScarlet. The time of nuclear envelope breakdown (NEBD) was set to zero; time is presented in minutes on each channel image. The white arrow indicates mitotic defects. Scale bars, 5 µm. DΔ: Δ248–253 and Δ280–300. H) Time from NEBD to anaphase onset was quantitated from the time‐lapse analysis shown in (G). I) Quantification of different types of mitotic defects from the same dataset as in (G). J) Colony formation of WT, CCDC68‐KO, and CCDC68‐KO HeLa cells stably expressing Flag‐CCDC68‐Full length (FL) or Flag‐CCDC68‐DΔ in the presence of DMSO or taxol (20 nm). Quantification of the clonogenic ratio. K) The cell growth rate was evaluated using the CCK‐8 assay. WT, CCDC68‐KO, and CCDC68‐KO HeLa cells stably expressing Flag‐CCDC68‐Full length (FL) or Flag‐CCDC68‐DΔ were treated with DMSO or taxol for the indicated times. All the data are presented as the means of the indicated biological replicates; error bars represent the means ± SEMs. Statistical analyses were performed using one‐way ANOVA for (B), (H), (J), and (K). n.s., not significant, *p < 0.05, **p < 0.01, ***p < 0.001, ****p < 0.0001.

Since CCDC68 was shown to maintain MCC integrity and restrict APC/C activity, we sought to examine the mechanism underlying the effect of CCDC68 on MCC integrity. CDC20 is continuously synthesized and degraded to achieve MCC turnover.^[^ ^2^, ^15^ ^]^ The rate of CDC20 proteolysis was significantly increased in CCDC68‐knockout HeLa cells compared with that in wild‐type cells (**Figure** **5**A,B). Consistently, the autoubiquitination of CDC20 was increased in CCDC68‐knockout cells that were arrested in mitosis (Figure 5C). Moreover, CCDC68‐Full length but not CCDC68‐DΔ restored the autoubiquitination of CDC20 in knockout cells that were arrested in mitosis (Figure 5D). CDC20 can be autoubiquitinated by APC/Cs that are purified from mitotic‐arrested cells.^[^ ^7^, ^15^, ^34^ ^]^ We then performed in vitro ubiquitination reactions supplemented with recombinant CCDC68 that was purified from bacteria, and we found that with the addition of the CCDC68 protein, the autoubiquitination of CDC20 was reduced (Figure 5E). When these reactions included APC/C that was purified from CCDC68‐depleted HeLa cells, CDC20 autoubiquitination level was higher than that from wild‐type cells (Figure 5F). Therefore, APC/C‐dependent CDC20 autoubiquitination is suppressed by CCDC68.

Next, we examined whether CCDC68‐mediated MCC integrity contributes to mitotic progression. First, flow cytometry analysis showed a decrease in G2/M cells and an increase in G1 cells following *CCDC68* knockout (Figure S7A, Supporting Information). Subsequently, we monitored cell division by live‐cell imaging using cells stably expressing H2B‐NeonGreen and α‐tubulin‐mScarlett. The time from the breakdown of the nuclear envelope to chromosome congression was decreased in CCDC68‐depleted cells. CCDC68‐Full length, but not CCDC68‐DΔ, rescued this decrease in the duration (Figure 5G,H; Figure S7B, Supporting Information). In contrast, the duration from chromosome congression to anaphase onset was not significantly altered by CCDC68 knockout (Figure S7B, Supporting Information), suggesting that CCDC68 regulates the duration specifically from nuclear envelope breakdown to congression. Moreover, expression of CCDC68‐Δ200–220, which lacks the ability to interact with HEC1 (Figure 1I), did not reverse the CCDC68 depletion‐induced decrease in the mitotic duration or increase in the proportion of cells with lagging chromosomes (Figure S7C,D, Supporting Information).

CCDC68‐knockout cells exhibited multiple mitotic defects, including an increase in incomplete congression, lagging chromosomes, and micronuclei, compared to wild‐type cells, which were all rescued by CCDC68‐Full length expression but not CCDC68‐DΔ expression (Figure 5I). Furthermore, we performed a colony formation assay to measure the survival ability of single cells and their potential to proliferate and form clonal populations. The results revealed that loss of CCDC68 attenuated tolerance to checkpoint abnormalities as well as taxol resistance (Figure 5J), and the clonogenic ratio was significantly restored by CCDC68‐Full length but not CCDC68‐DΔ (Figure 5J). The growth rate of CCDC68‐knockout cells was reduced, as shown by the CCK‐8 assay (Figure 5K), and this effect was rescued by CCDC68‐Full length but not CCDC68‐DΔ (Figure 5K). Resistance to taxol was also attenuated in CCDC68‐knockout cells, and CCDC68‐Full length, but not the CCDC68‐DΔ, completely rescued the decrease in drug resistance (Figure 5K). Thus, CCDC68 contributes to faithful chromosome segregation by maintaining MCC integrity.

### 2.6 Downregulation of CCDC68 Induces Aneuploidy

**Figure 6:**
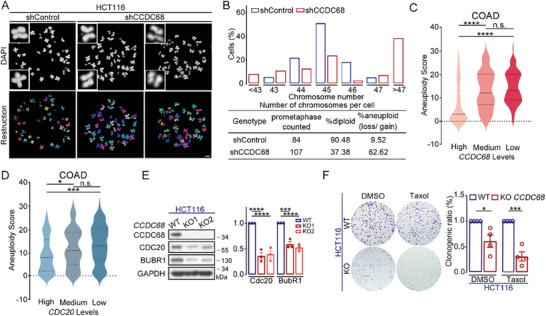
Downregulation of CCDC68 induces aneuploidy. A) Images of DAPI‐stained mitotic spread in shControl or shCCDC68 HCT116 cells (upper panel). Schematic representation of the 3D reconstruction of chromosome spread (lower panel). Scale bar: 5 µm. B) Quantification of the distribution of the total chromosome number and the percentage of diploid and aneuploid cells shown in (A). C,D) Bioinformatics analysis of aneuploidy scores in association with CCDC68 mRNA expression (C) and CDC20 mRNA expression (D) in colon cancer patients. The mRNA expression data were downloaded from the TCGA database (https://www.cbioportal.org/). COAD: colon adenocarcinoma. E) Immunoblotting of lysates from wild‐type (WT) and CCDC68‐knockout (KO) HCT116 cells. The cells were treated with nocodazole for 16 h then released to fresh media for 30 min. GAPDH served as the loading control. Quantification of the relative intensity. F) Colony formation of WT and CCDC68‐KO HCT116 cells in the presence of DMSO or Taxol (20 nm). Quantification of the clonogenic ratio. All the data are presented as the means of the indicated biological replicates; error bars represent the means ± SEMs. Statistical analyses were performed using Student's *t*‐test for (F) and using one‐way ANOVA for (C–E). n.s., not significant. *p < 0.05, **p < 0.01, ***p < 0.001, ****p < 0.0001.

Chromosome mis‐segregation causes a change in karyotype and results in aneuploidy in daughter cells after cell division. To further investigate the roles of CCDC68 in genomic stability, we enumerated the chromosomes in HCT116 cells by chromosome spread assay (**Figure** **6**A). After the knockdown of CCDC68, the number of cells containing >46 chromosomes increased by approximately fourfold (Figure 6A,B), suggesting that depletion of CCDC68 causes aneuploidy. We also observed a gap between the sister chromatids at the centromere in the CCDC68‐depleted cells (Figure 6A), which resulted in defective cohesion. Furthermore, we used the SunTag system, which efficiently recruits multiple proteins to a single genomic locus to amplify the signal by enhancing transcriptional activation.^[^ ^36^ ^]^ Following coexpression of *Myc*‐sgRNA, dCas9‐10×GCN4v4 and scFv‐sfGFP, the copy number of *Myc* was labeled. The percentage of cells containing more than 2 *Myc* dots was increased to ≈60% in CCDC68‐knockout HeLa cells compared with ∼30% in wild‐type cells (Figure S8A,B, Supporting Information), further showing that the deficiency of CCDC68 leads to aneuploidy.

An aneuploidy score was calculated in 10522 samples spanning 33 cancer types from The Cancer Genome Atlas (TCGA) pan‐cancer dataset,^[^ ^37^ ^]^ allowing us to correlate gene expression to aneuploidy. Our analysis revealed that lower mRNA expression of CCDC68 was significantly associated with higher aneuploidy in colorecal, breast, and lung carcinomas (Figure 6C; Figure S8C,D, Supporting Information). Moreover, the mRNA expression levels of CDC20 and MAD2 also exhibited a negative correlation with the aneuploidy score in colorectal cancer (Figure 6D; Figure S8E, Supporting Information). HCT116 is a type of cancer cell with a relatively low aneuploidy rate. We observed that the protein levels of CDC20 and BUBR1 were decreased by ≈50% in CCDC68‐knockout HCT116 cells during late prometaphase (Figure 6E; Figure S8F–J, Supporting Information), suggesting that CDC20 and BUBR1 protein levels were at least partly maintained by CCDC68 in cancer cells with low aneuploidy rates. CCDC68‐knockout HCT116 cells showed a reduced clonogenic ratio and attenuated resistance to taxol (Figure 6F), further suggesting that CCDC68 contributes to cell survival and SAC activation in cancer cells with low aneuploidy rates. Therefore, a low abundance of CCDC68 correlates with a high risk of aneuploidy.

To further assess the involvement of CCDC68 in tumor growth regulation, we injected wild‐type and CCDC68‐knockout HCT116 cells into nude mice to establish colorectal cancer xenograft mouse models (Figure S8K, Supporting Information). The tumor growth rate was significantly lower in mice injected with HCT116 CCDC68‐knockout cells than in mice injected with wild‐type cells (Figure S8L–N, Supporting Information). Consistently, the loss of CCDC68 in HCT116 cells increased survival of mice (Figure S8O, Supporting Information). These results suggest that the loss of CCDC68 reduces the tumor growth rate by perturbing mitotic progression.

## 3 Discussion

**Figure 7:**
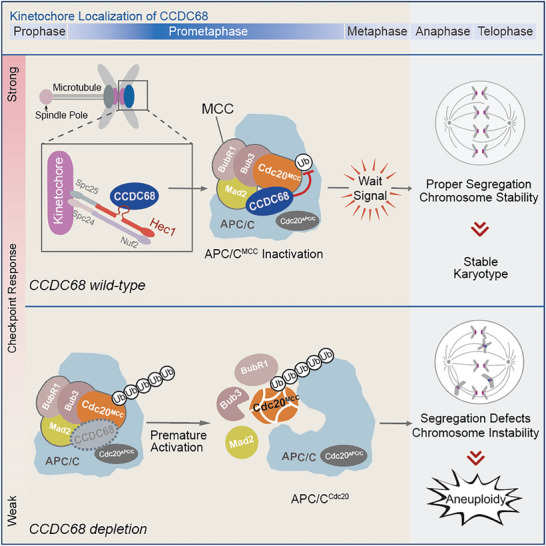
Schematic diagram showing that CCDC68 ensures proper chromosome segregation by promoting CDC20 integration into the MCC. CCDC68 is recruited to unaligned kinetochores during prometaphase. CCDC68 interacts with CDC20 and MAD2 to inhibit the autoubiquitination of CDC20 and restrains the turnover of the MCC on the APC/C, thus maintaining the strength of the SAC and the fidelity of chromosome alignment.

In this study, we found that CCDC68 is an outer kinetochore protein that recognizes unaligned kinetochores to promote SAC activation. Mechanistically, CCDC68 binds directly to CDC20 and MAD2 to maintain CDC20 equilibrium and MCC integrity, which guarantees proper chromosome alignment and segregation by preventing premature chromosome segregation (**Figure** **7**).

The accumulation of CCDC68 at kinetochores is highly dynamic; it begins in prophase, peaks in prometaphase, and decreases as chromosomes are aligned and segregated. CCDC68 directly interacts with CDC20 and MAD2 to restrict the autoubiquitination of CDC20, and fully attached kinetochores exclude the localization of CCDC68 and dismiss its stabilization effect on CDC20. CCDC68 promotes the integrity of the MCC to inhibit the APC/C carrying out ubiquitination and degradation of substrates.

Unlike most outer kinetochore proteins that localize to kinetochores from prophase to telophase, CCDC68 selectively localizes to unaligned kinetochores prior to anaphase onset (Figure 1C), which provides an additional mechanism that underlies the surveillance of chromosome alignment. The surveillance of chromosome alignment is mediated by multiple kinetochore proteins, such as BUBR1,^[^ ^3^ ^]^ BUB1,^[^ ^5^ ^]^ BUB3,^[^ ^4^ ^]^ MAD2,^[^ ^7^, ^38^ ^]^ and RZZ.^[^ ^6^ ^]^ However, most of these proteins persist at kinetochores when anaphase onset, except for MAD2 and RZZ, which can be transported away from kinetochores by the dynein complex.^[^ ^39^ ^]^ As a factor that interacts with MAD2 (Figure 3A,C), the disassociation of CCDC68 from kinetochores once the kinetochores are attached by spindle microtubules may also be driven by the dynein complex, but this needs further investigation.

Rapid MCC turnover is promoted by continuous cycles of CDC20 synthesis and degradation during prometaphase.^[^ ^15^ ^]^ APC15 and TRIP13 have been reported to be two catalytic pathways that inactive the MCC.^[^ ^18^ ^]^ APC15 is a subunit of the APC/C complex and is responsible for CDC20 autoubiquitination and subsequent proteasomal degradation, which triggers MCC disassembly.^[^ ^14^, ^15^ ^]^ TRIP13/p31^comet^ catalyzes the removal of MAD2 to disassemble the MCC.^[^ ^18^ ^]^ However, there is no reported mechanism for the positive regulation of the MCC. Here, we reveal that CCDC68 functions as a positive regulator of MCC integrity to prevent the autoubiquitination of CDC20, providing new insight into the mechanisms by which the SAC is regulated.

A functional spindle assembly checkpoint (SAC) is crucial for viability in eukaryotes. Mice lacking MAD2 experience embryonic lethality at the blastocyst stage.^[^ ^40^ ^]^ Cdc20‐deficient mice also exhibit embryonic lethality due to metaphase arrest resulting from securin stabilization.^[^ ^41^ ^]^ Whether the viability of Ccdc68‐knockout mice is compromised requires further investigation. Given the fact that loss of CCDC68 compromises cell viability in xenograft mouse models, it is evident that CCDC68 is one of the key factors that contributes to the maintenance of genomic stability.

## 4 Experimental Section

### Plasmid Construction

The full‐length complementary DNAs (cDNA) of CCDC68, HEC1, CDC20, securin, H2B, and α‐tubulin were amplified from HEK293T cell cDNA by PCR. The full‐length and truncated cDNAs were cloned and inserted into pCMV7.1‐3×Flag, pCDNA3.1‐HA, pCDNA3.1‐V5, pEGFP‐C2, pEGFP‐N3, pGEX‐6P‐1, pSin‐3×Flag, pSin‐NeonGreen, pSin‐mScarlett, pSin‐mEmerald, or pCold‐MBP.

### Cell Culture, Transfection, and Treatment

HEK293T, HeLa, RPE‐1, and HCT116 cells were purchased from and validated by ATCC. HEK293T, HeLa, and HCT116 cells were cultured in DMEM (Gibco) supplemented with 10% fetal bovine serum (CellMax) at 37 °C in a humidified atmosphere of 5% CO_2_. RPE‐1 cells were cultured in F12/DMEM (Gibco) supplemented with 10% fetal bovine serum (CellMax). HEK293T cells were transfected using PEI (Polysciences, Inc., 23966‐1). HeLa cells were transfected using Lipofectamine 3000 (Invitrogen) for 24 h, according to the manufacturer's instructions.

For collecting cells in prometaphase for immunoprecipitation, cells were treated with nocodazole (100 ng mL^−1^, Sigma) for 20 h or taxol (20 nm, Selleckchem) for 24 h to synchronize them. For activating the spindle assembly checkpoint, cells were synchronized with thymidine (2 mm, Sigma) and released in taxol (5 nm, Selleckchem). For collecting cells in prometaphase for immunofluorescence, cells were treated with monastrol (100 µm, Sigma) for 5 h or synchronized with thymidine and released in taxol (20 nm). For isolating cells from different stages, cells were synchronized by treatment with double‐thymidine (2 mm) and then released in fresh medium for 9–10 h. For inducing the unaligned chromosomes, cells were treated with GSK923295 (20 mM, Cayman) for 2 h prior to fixation.

### RNA Interference

The sense‐strand sequence of the negative control siRNA was 5′‐UUCUCCGAACGUGUCACGUTT‐3′. The sense‐strand sequence HEC1 siRNA was 5′‐ AAGUUCAAAAGCUGGAUGAUCUU −3′. The sense‐strand sequence of CCDC68 siRNA‐1 was 5′‐ CUGCGUGAGUCUUAUUUAUTT −3′. The sense‐strand sequence of CCDC68 siRNA‐2 was 5′‐ GGCUGUCUCUACAAGUGAAUU −3′. The sense‐strand sequence of MAD2 siRNA‐1 was 5′‐ GGAAGAGUCGGGACCACAGUU −3′. The sense‐strand sequence of MAD2 siRNA‐2 was 5′‐ GUGCAGAAAUACGGACUCAUU −3′. The sense‐strand sequence of CDC20 siRNA‐1 was 5′‐ CGGAAGACCUGCCGUUACAUU −3′. The sense‐strand sequence of CDC20 siRNA‐2 was 5′‐ UGCGCCUGAAAUCCGAAAUUU −3′. The siRNAs were synthesized by GenePharma, and siRNA transfection experiments were performed using Lipofectamine 3000 (Invitrogen) for 48 h or 24 h, according to the manufacturer's instructions. The siRNA‐resistant cDNA was cloned by overlap extension PCR.

### GST Pull‐Down, Immunoprecipitation, and Immunoblotting

For GST pull‐down assays, GST‐tagged and MBP‐tagged fusion proteins were expressed in *Escherichia coli* BL21 cells. For expressing GST‐tagged and MBP‐tagged full‐length or mutant proteins, BL21 cells in the logarithmic phrase of growth were treated by the addition of 1 mM IPTG and incubated overnight at 16 °C. Harvested cells were ultrasonically lysed in pull‐down buffer (0.5% Tween‐20, 20 mm Tris‐HCl, 200 mm NaCl, 1 mm DTT, 5 mm EGTA, pH 7.5), and the GST fusion proteins were purified using Glutathione Sepharose 4B beads (GE Healthcare). For obtaining the proteins for the in vitro pull‐down assay, GST‐tagged proteins bound to Glutathione Sepharose Resin were eluted by the addition of GSH (10 mm), and MBP‐tagged proteins bound to Amylose Beads (GE Healthcare) were eluted with MBP‐fusion elution buffer (pull‐down buffer containing 10 mm maltose) for 1 h at 4 °C with agitation. Proteins for the in vitro assay were eluted by cleaving their GST‐tag or MBP‐tag using PreScission protease overnight at 4 °C (Genscript, Z02799). HEK293T cells that were transfected with the indicated plasmids were lysed in cell lysis buffer (1% NP40, 50 mm Tris‐HCl, 200 mM NaCl and 10% glycerol, pH 7.4) containing protease inhibitor cocktail. Lysates were cleared by centrifugation at 12000 × g for 10 min and then incubated with GST‐fusion protein‐bound beads for 2 h at 4 °C. The beads were washed five times with cell lysis buffer, resuspended in protein loading buffer (50 mm Tris‐HCl, 2% SDS, 100 mm DTT, 10% glycerol and 0.025% bromophenol blue, pH 6.8), and boiled for 10 min. Protein samples were separated by SDS‒PAGE and analyzed by immunoblotting with the indicated antibodies or Coomassie Blue staining.

For coimmunoprecipitation, cells were cultured in 10 cm dishes and lysed on ice in lysis buffer (1% NP‐40, 50 mm Tris‐HCl 150 mm NaCl, and 10% glycerol, pH 7.4) containing protease inhibitor cocktail. Cell debris was pelleted by centrifugation at 12000 × g for 15 min at 4 °C, and the lysates were incubated with the indicated antibodies for 2 h at 4 °C and then with Protein G Sepharose beads (GE Healthcare) for an additional 2 h. The immunoprecipitated samples were boiled in protein loading buffer and analyzed by immunoblotting.

For immunoblotting, protein samples were separated by SDS‒PAGE and transferred to polyvinylidene difluoride membranes (Millipore). The membranes were blocked and then probed with the following primary antibodies: anti‐Flag (Sigma‒Aldrich, F1804, 1:1000), anti‐GAPDH (CWBIO, 0100A, 1:5000), anti‐α‐tubulin (Abcam, ab11304, 1:2000), anti‐HA (Sigma‒Aldrich, H9685, 1:1000), anti‐V5 (Innovative Research, R960CUS, 1:1000), anti‐ubiquitin (Proteintech, 10201‐2‐AP, 1:500), anti‐CCDC68 (GeneTex, GTX106883, 1:100), anti‐cyclin E2 (Cell Signaling Technology, 4132S, 1:500), anti‐cyclin B1 (Cell Signaling Technology, 4138S, 1:500), anti‐pH3 (Cell Signaling Technology, 9701S, 1:500), anti‐HEC1 (Abcam, ab3613, 1:500), anti‐BUBR1 (GeneTex, GTX111289, 1:500), anti‐BUB1 (GeneTex, GTX107494, 1:100), anti‐BUB3 (GeneTex, GTX113595, 1:500), anti‐CDC20 (Santa Cruz Biotechnology, sc‐13162, 1:400), anti‐APC3 (Santa Cruz Biotechnology, sc‐9972, 1:300), anti‐APC2 (Proteintech, 13559‐1‐AP, 1:500), anti‐APC5 (Proteintech, 67348‐1‐Ig, 1:500), anti‐APC4 (Proteintech, 14129‐1‐AP, 1:500), anti‐APC8 (Proteintech, 10683‐1‐AP, 1:200), anti‐securin (EPITOMICS, 2603‐S, 1:500), anti‐MAD2L1 (Proteintech, 10337‐1‐AP, 1:500), anti‐NUF2 (Proteintech, 15731‐1‐AP, 1:500), and anti‐GFP (Abcam, ab290, 1:1000) antibodies. The membranes were washed in TBS containing 0.05% Tween‐20, and probed with peroxidase‐Affinipure goat anti‐rabbit or anti‐mouse IgG (H+L) secondary antibodies (Jackson ImmunoResearch, 111‐035‐003 and 115‐035‐003, 1:5000) for 2 h at room temperature. The membrane was exposed using a film processing instrument (Kodak) or an Amersham Imager 600 (GE Healthcare).

### Immunofluorescence

For kinetochore localization experiments, cells were fixed and pre‐extracted at 37 °C for 90 s in PEMGT (100 mm PIPES, pH 6.9, 10 mm EGTA, 1 mm MgCl_2_, 4 M glycerol, and 0.5% Triton X‐100) prior to incubation for 10 min in PFA at 37 °C. For the chromosome spread assay, suspended mitotic cells were centrifuged on a coverslip at 1500 × g for 3 min after incubation with 0.4% KCl solution at room temperature for 20 min. Then, the cells were fixed at −20 °C for 10 min in cold methanol. The cells were blocked and then probed with primary antibodies in 4% BSA, washed with PBS containing 0.1% Triton X‐100, and probed with Alexa 488‐ or 561‐conjugated secondary antibodies (Thermo Fisher Scientific). The cells were finally stained with DAPI (1 µg mL^−1^ 4,6‐diamidino‐2‐phenylindole) prior to imaging. Immunostaining was performed using the following antibodies: anti‐Flag (Sigma‒Aldrich, F1804, 1:200), anti‐α‐tubulin (Abcam, ab11304, 1:500), anti‐CCDC68 (Proteintech, 26301‐1‐AP, 1:100), anti‐ACA (ImmunoVision, HCT0100, 1:100), anti‐CENP‐A (MBL, D115‐3,1:250), anti‐CENP‐C (Abcam, ab50974, 1:100), anti‐Centrin 3 (Abnova, H00001070‐M01, 1:200), anti‐HEC1 (Abcam, ab3613, 1:100), anti‐MAD1 (Santa Cruz Biotechnology, sc‐47746, 1:50), anti‐MIS12 (Abcam, ab70843, 1:100), anti‐BUBR1 (Thermo Fisher, MA1‐16577, 1:50), anti‐MAD2L1 (Santa Cruz Biotechnology, sc‐47747, 1:50) anti‐BUB1 (GeneTex, GTX107494, 1:100), anti‐CDC20 (Santa Cruz Biotechnology, sc‐13162, 1:50), and anti‐ZW10 (Abcam, ab53676, 1:100) antibodies. All the samples were observed under a confocal microscope at room temperature. Image processing was performed using Photoshop (2020, Adobe). Briefly, the raw images were projected and exported as tiff files and further analyzed using Imaris (9.6, Oxford Instruments) or ImageJ (National Institutes of Health) software. For quantifying the relative intensity of kinetochore components, kinetochore regions were selected based on the CENP‐A or ACA signal, and an area of the same size was selected in nonkinetochore regions for background determination. The relative kinetochore intensity of a given protein was calculated as the mean of its kinetochore intensity after the background intensity was subtracted divided by the kinetochore intensity of CENP‐A or ACA after the background intensity was subtracted.

### Generation of CCDC68‐Knockout and Stable Expressing Cell Lines

All knockout cell lines were generated using the CRISPR/Cas9 approach. The sgRNAs were designed using the online webtool (chopchop.cbu.uib.no) and cloned and inserted into the lentiCRISPR v2 vector. HEK293T cells were transfected with lentiCRISPR v2 and plasmids for virus packaging (psPAX2 and pMD2.G). For obtaining viruses, cells were cultured for an additional 48 h, and medium containing viruses was cleared with a 0.22‐µm filter and concentrated with PEG8000. HeLa cells were infected with virus for 48 h in the presence of 8 µg mL^−1^ polybrene. Puromycin‐resistant cells were selected, and colonies were grown from single cells. The colonies were subjected to immunoblotting, immunofluorescence, and genotyping to verify the successful knockout of CCDC68. Cells with no “‘peak‐on‐peak”’ sequences were considered to have the same edit in all their genome copies. HeLa cells stably expressing Flag‐CCDC68‐Full length, Flag‐CCDC68‐DΔ, H2B‐NeonGreen, or α‐tubulin‐mScarlett were generated using a lentiviral system. The plasmids for stable expression were generated using the pSin vector. The procedure used to obtain the virus was the same as that described above.

### Time‐Lapse Microscopy

For high spatiotemporal resolution time‐lapse imaging, 2‐µm‐separated z‐planes covering the entire volume of mitotic cells were collected every 4 min as indicated. Time‐lapse imaging was performed using an inverted confocal microscope (Dragonfly, Leica DMI8) or a PerkinElmer UltraView Vox spinning‐disk confocal microscope and Volocity software. The cells were cultured in a 37 °C and 5% CO_2_ environmental chamber on the microscope stage with a lens‐heating collar. Images were postprocessed using Imaris (9.6, Oxford Instruments) or Volocity (Nikon) software.

### CDC20 Autoubiquitination by the APC/C In Vitro

The method for in vitro CDC20 autoubiquitination was adopted from other studies,^[^ ^7^, ^15^ ^]^ with the following changes. In brief, CCDC68 was depleted by siRNA treatment for 48 h before the APC/C was purified from mitotic HeLa cell extracts with an anti‐APC3 antibody. Immunoprecipitates were resuspended with purified CDC20 and CCDC68 in conjunction with 0.5 µm E1, 10 µm E2 (UbcH10), 1.5 mg mL^−1^ ubiquitin, ATP‐regenerating system (7.5 mm creatine phosphate, 10 mm ATP, and 2 mm MgCl_2_), 50 mm PIPES, 1 mm dithiothreitol, 10% glycerol, and 1 mm EGTA. The reactions were incubated at 37 °C for the indicated times, separated by SDS‒PAGE, and subjected to immunoblotting.

### Flow Cytometry

Cells were washed twice with cold PBS and fixed in cold methanol at −20 °C overnight. The fixed cells were washed twice with PBS, treated with 100 mg mL^−1^ RNase A at 37 °C for 30 min, stained with propidium iodide for an additional 30 min, and analyzed using FACSVerse (BD Biosciences) and FlowJo (BD Biosciences) software.

### Cell Viability Assay

A total of 1 × 10^4^ cells were seeded in each well of a 96‐well plate. After being cultured for 24 h, the cells were treated with taxol (20 nM) or DMSO for 36 h or 72 h. At each time point, an CCK‐8 assay (DOJINDO, CK04) was performed, and the absorbance was measured at a wavelength of 490 nm using an absorbance microplate reader (TECAN, Sunrise).

### Clonogenicity Assay

Cells were treated with DMSO or taxol (Selleck, S1150) for 36 h and subsequently trypsinized and counted after trypan blue staining using a LunaII (Logos Biosystems, L40002). A total of 1 × 10^6^ cells were seeded in each well of a 6‐well plate. After 10 d, the cells were fixed with 4% PFA for 10 min at 37 °C, stained with crystal violet solution (Beyotime, C1021), destained with tap water, and dried in an oven.

### Chromosome Spread Assay

Cells were treated with nocodazole or taxol for 16–24 h to collect cells in prometaphase. After shaking the plate, the cell suspension was centrifuged for 5 min at 300 × g, gently resuspended in 0.4% KCl solution, and incubated at room temperature for 20 min. Freshly prepared methanol:acetic acid (3:1) fixative buffer was added, and the cells were incubated at −20 °C for 10 min. The cell suspension was dropped onto a precooled glass slide from a height of 1 m. After the slides were dried, DNA was stained with DAPI. The cells were imaged using a confocal microscope and counted using Imaris software (9.6, Oxford Instruments).

### Subcutaneous Xenograft Tumor Growth

All animals were handled following the “Principles for the Utilization and Care of Vertebrate Animals” and the “Guide for the Care and Use of Laboratory Animals”. Animal studies were approved by the IACUC of the Center for Experimental Animal Research (China) and Peking University Laboratory Animal Center (IACUC No. LSC‐ChenJG‐3). Female BALB/c nude mice (6–8 weeks of age) were purchased from Beijing Vital River Laboratory Animal Technology. Wild‐type and CCDC68‐knockout HCT116 cells were collected and counted after trypan blue staining to exclude dead cells. Each cell suspension containing 1 × 10^6^ cells was mixed with Matrigel (ABW, 082726) at a 1:1 ratio to obtain a final injection volume of 200 µL. The mixture was injected into the right armpit of BALB/c nude mice using an insulin syringe (BD Biosciences, 1 mL). Tumors were measured every 5 days using an electric caliper, and tumor volume was calculated (width^2^ × length × 0.5). On the last day, the xenograft tumors were dissected and weighed. All tumors were kept under 15 mm at their largest dimension; none of the xenograft tumors exceeded this limit. Mice were chosen in an unbiased manner for randomization.

### Statistical Analysis

Statistical analysis was performed using Prism 9.0 (GraphPad Software). Student's *t*‐test was used for comparisons between two groups. For comparisons among multiple groups, one‐way ANOVA was used. All the experiments were performed at least three times. *p < 0.05, **p < 0.01, ***p < 0.001, ****p < 0.0001, n.s., not significant.

## Conflict of Interest

The authors declare no conflict of interest.

## Author Contributions

Q.L. designed and performed most of the molecular biology experiments, cell biology experiments, and data analysis. Q.C., T.Z., and F.W. constructed several plasmids and provided insightful advice. J.T., H.Z., and J.C. are the senior authors who designed the project. Q.L., J.T., H.Z., and J.C. co‐wrote the manuscript.

## Supporting information

Supporting Information

## Data Availability

The data that support the findings of this study are available from the corresponding author upon reasonable request.
