# Supplementary material for: CCDC68 Maintains Mitotic Checkpoint Activation by Promoting CDC20 Integration into the MCC
Source: Adv Sci (Weinh). 2024 Jul 17;11(35):2406009. doi: 10.1002/advs.202406009 (PMC11425217; doi:10.1002/advs.202406009)
Supplement: Supplementary file 1 — Supporting Information [file ADVS-11-2406009-s001.docx]

**Supplemental Information**

**CCDC68 Maintains Mitotic Checkpoint Activation by Promoting CDC20 Integration into the MCC**

Qi Li, Tao Zheng, Qingzhou Chen, Fulin Wang, Junlin Teng, Haining Zhou, Jianguo Chen

**Supplementary information, Fig. S1. CCDC68 is localized to the outer plate of unaligned kinetochores by interacting with HEC1. Related to Fig. 1.**


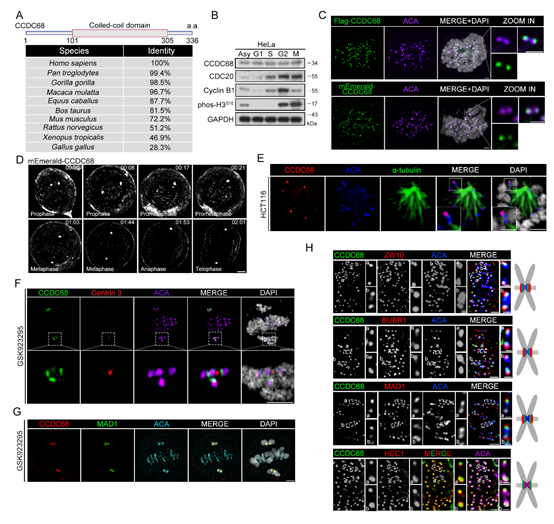


(**A**) Schematic and homology analysis of CCDC68. (**B**) Immunoblotting of lysates from HeLa cells at different stages in the cell cycle. GAPDH served as the loading control. (**C**) Immunofluorescence staining for ACA (purple) in HeLa cells transfected with Flag-CCDC68 (green, upper) or mEmerald-CCDC68 (green, lower). Scale bar in the full image, 2 μm; scale bar in the cut-out, 1 μm. (**D**) Live-cell imaging of HeLa cells transfected with mEmerald-CCDC68. The time of nuclear envelope breakdown was set to zero. Scale bar, 5 μm. (**E**) Immunofluorescence staining for CCDC68 (red), α-tubulin (green), and ACA (blue) in monastrol-treated HCT116 cells. DNA was stained with DAPI (white). Scale bar in the full image, 2 μm; Scale bar in the cut-out, 0.5 μm. (**F**) Immunofluorescence staining for CCDC68 (green), Centrin 3 (red), and ACA (purple) in HeLa cells treated with GSK923295 for 3 h. DNA was stained with DAPI (white). An unaligned kinetochore is included in the dotted box at the spindle poles. Scale bar in the full image, 2 μm; Scale bar in the cut-out, 1 μm. (**G**) Immunofluorescence staining for CCDC68 (red), MAD1 (green), and ACA (cyan) in HeLa cells treated with GSK923295 for 3 h. DNA was stained with DAPI (white). Scale bars, 2 μm. (**H**) HeLa cells were treated with MG132 for 1 h and subsequently subjected to immunostaining for CCDC68 (green), ZW10 (red, first panel), BUBR1 (red, second panel), MAD1 (red, third panel), and HEC1 (red, fourth panel). ACA (purple or blue) was stained as an inner kinetochore marker. Scale bar in the full image, 2 μm; Scale bar in the cut-out, 0.5 μm.

**Supplementary information, Fig. S2. CCDC68 is localized to the outer plate of unaligned kinetochores by interacting with HEC1. Related to Fig. 1.**


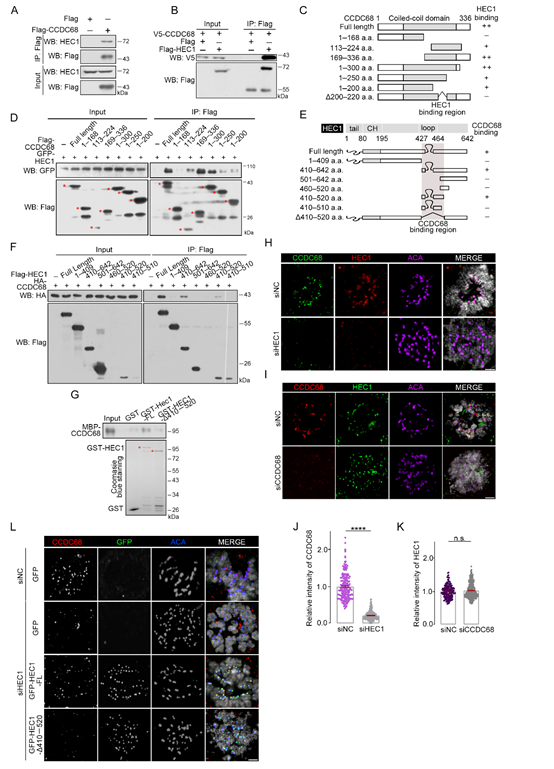


(**A**) Lysates from HEK293T cells overexpressing Flag-CCDC68 were subjected to immunoprecipitation (IP) with an anti-Flag antibody, and the samples were analyzed by immunoblotting with the indicated antibodies. (**B**) HEK293T cells coexpressing Flag-vector or Flag-HEC1 and V5-CCDC68 were subjected to IP with an anti-Flag antibody, and the resulting samples were analyzed by immunoblotting with the indicated antibodies. (**C**) Schematic diagram of full-length CCDC68, truncated CCDC68 mutants, and CCDC68 deletion mutants. Hec1 binding ability: +, positive; -, negative. (**D**) HEK293T cells coexpressing Flag-empty or Flag-CCDC68 truncation mutants and GFP-HEC1 were subjected to IP with an anti-Flag antibody and analyzed by immunoblotting with the indicated antibodies. (**E**) Schematic diagram of full-length HEC1, the HEC1 truncation mutants, and the HEC1 deletion mutants. CCDC68 binding ability: +, positive; -, negative. (**F**) HEK293T cells coexpressing Flag-vector or the Flag-HEC1 truncation mutant and HA-CCDC68 were subjected to IP with an anti-Flag antibody and analyzed by immunoblotting with the indicated antibodies. (**G**) MBP-CCDC68, GST-HEC1-Full length (FL), and GST-HEC1-Δ410–520 were purified in *E. coli* and subjected to *in vitro* pull-down assays. MBP-CCDC68 was incubated separately with GST-HEC1-Full length (FL) and GST-HEC1-Δ410–520, and the samples were analyzed by immunoblotting with an anti-CCDC68 antibody and Coomassie blue staining. (**H**) Immunofluorescence staining for CCDC68 (green), HEC1 (red), and ACA (purple) in HeLa cells transfected with siNC or HEC1 siRNA. DNA was stained with DAPI (white). Scale bar, 2 μm. NC, negative control. (**I**) Immunofluorescence staining for CCDC68 (red), HEC1 (green), and ACA (purple) in HeLa cells transfected with siNC or CCDC68 siRNA. DNA was stained with DAPI (white). Scale bar, 2 μm. NC, negative control. (**J**) Quantification of the relative intensity of CCDC68 staining on kinetochores. More than 100 kinetochores from 10 cells were analyzed in (**H**). (**K**) Quantification of the relative intensity of HEC1 staining on kinetochores. More than 100 kinetochores from 10 cells were analyzed in (**I**). (**L**) Immunofluorescence staining of HeLa cells cotransfected with GFP-HEC1-Full length (FL) or GFP- HEC1-Δ410–520 and HEC1 siRNA. Cells were stained for CCDC68 (red), GFP (green) and ACA (blue). DNA was stained with DAPI (white). Scale bars in the full image, 2 μm; scale bars in the cut-out, 0.5 μm. All the data are presented as the means of the indicated biological replicates; error bars represent the means ± SEMs. Statistical analyses were performed using Student’s t test for (**J**) and (**K**). n.s., not significant, *****P* < 0.0001.

**Supplementary information, Fig. S3. CCDC68 depletion accelerates anaphase onset and leads to chromosome lagging. Related to Fig. 2.**


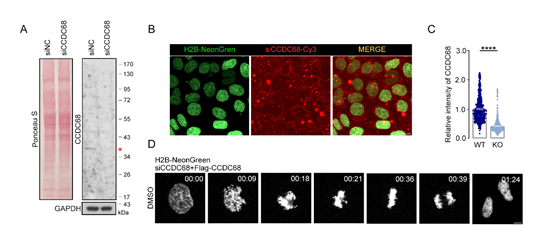


(**A**). Immunoblotting of lysates from HeLa cells transfected with siNC or CCDC68 siRNA. GAPDH and Ponceau S staining served as the loading controls. (**B**). Images of HeLa cells stably expressing H2B-NeonGreen and transfected with siCCDC68-Cy3. Scale bar, 5 μm. (**C**) Quantification of the fluorescence intensity of CCDC68 on kinetochores shown in Figure **2C**. More than 100 kinetochores from 10 cells were analyzed. (**D**) Live-cell imaging of HeLa cells transfected with siCCDC68 and Flag-CCDC68-resistant (Flag-CCDC68). The cells also stably expressed H2B-NeonGreen. The time of nuclear envelope breakdown (NEBD) was set to zero; time is presented in minutes on each channel image. Scale bars, 5 μm. All the data are presented as the means of the indicated biological replicates; error bars represent the means ± SEMs. Statistical analyses were performed using Student’s t test for (**C**) *****P* < 0.0001.

**Supplementary information, Fig. S4. CCDC68 stabilizes MCC by interacting with CDC20 and MAD2. Related to Fig. 3.**


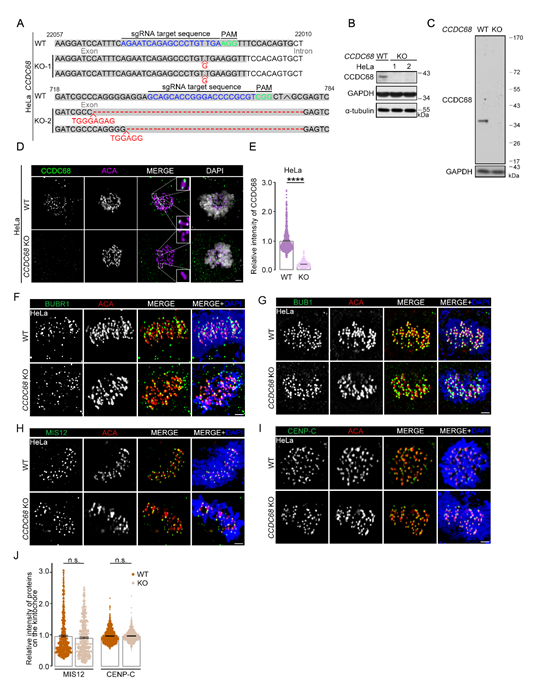


(**A**) Schematic of wild-type (WT) and *CCDC68*-knockout (KO) HeLa cell lines. The protospacer adjacent motif (PAM) sequence is highlighted in green, the sgRNA target sequence is highlighted in blue, and the mutated sequence is highlighted in red. The sgRNA deleted the exon‒intron splice site, which was accompanied by frameshift indels in the targeted region. (**B**) Immunoblotting of lysates from wild-type (WT) HeLa and *CCDC68*-KO HeLa cells. GAPDH and α-tubulin served as the loading controls. (**C**) Immunoblotting of lysates from WT HeLa, *CCDC68*-KO HeLa, and *CCDC68*-KO HeLa cells expressing Flag-CCDC68. GAPDH served as the loading control. (**D**) Immunostaining for CCDC68 (green) and ACA (purple) in WT or *CCDC68* KO cells. DNA was stained with DAPI (white). Scale bar, 2 μm. (**E**) Quantification of the fluorescence intensity of CCDC68 on the kinetochores shown in (**D**). More than 100 kinetochores from 10 cells were analyzed. (**F–I**) Immunostaining for BUBR1 (green), BUB1 (green), MIS12 (green), or CENP-C (green) and ACA (red) in wild-type (WT) or *CCDC68*-knockout (KO) HeLa cells. DNA was stained with DAPI (blue). Scale bar, 2 μm. (**J**) Quantification of the relative intensity of MIS12 (**H**) and CENP-C (**I**) staining on kinetochores. More than 100 kinetochores from 10 cells were analyzed. All the data are presented as the means of the indicated biological replicates; error bars represent the means ± SEMs. Statistical analyses were performed using Student’s t test for (**E**) and (**J**). n.s., not significant, *****P* < 0.0001.

**Supplementary information, Fig. S5. CCDC68 stabilizes MCC by interacting with CDC20 and MAD2. Related to Fig. 3.**


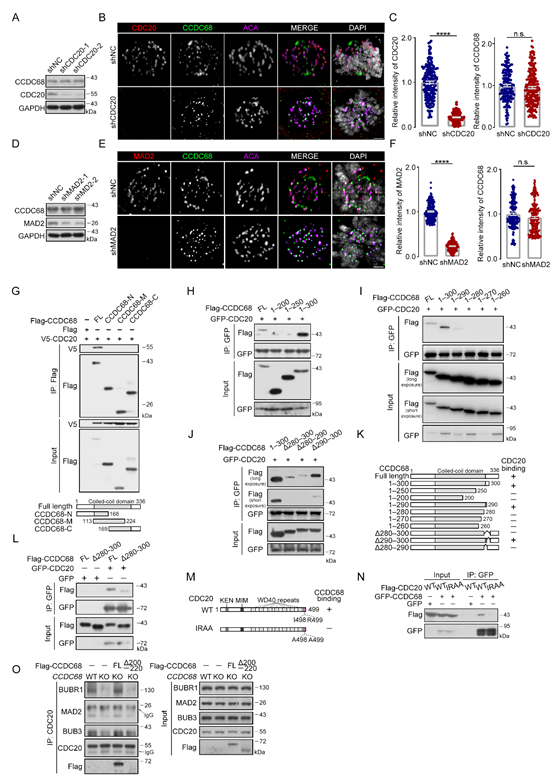


(**A**) Immunoblotting of lysates from HeLa cells transfected with shNC, shCDC20-1, and shCDC20-2. GAPDH served as the loading control. (**B**) Immunostaining for CDC20 (red), CCDC68 (green), and ACA (purple) in HeLa cells transfected with shNC or shCDC20. DNA was stained with DAPI (white). Scale bar, 2 μm. (**C**) Quantification of the relative intensity of CDC20 and CCDC68 staining on kinetochores. More than 100 kinetochores from 10 cells were analyzed. (**D**) Immunoblotting of lysates from HeLa cells transfected with shNC, shMAD2-1, or shMAD2-2. GAPDH served as the loading control. (**E**) Immunostaining for MAD2 (red), CCDC68 (green), and ACA (purple) in HeLa cells transfected with shNC or shMAD2. DNA was stained with DAPI (white). Scale bar, 2 μm. (**F**) Quantification of the relative intensity of MAD2 and CCDC68 staining on kinetochores. More than 100 kinetochores from 10 cells were analyzed. (**G**) HEK293T cells coexpressing Flag-empty, Flag-CCDC68-Full length (FL), or Flag-CCDC68-N/M/C and V5-CDC20 were subjected to immunoprecipitation (IP) with an anti-Flag antibody and analyzed by immunoblotting with the indicated antibodies. Schematic diagram of CCDC68-Full length (FL), CCDC68-N, CCDC68-M, and CCDC68-C. (**H**) HEK293T cells coexpressing Flag-CCDC68-Full length (FL), Flag-CCDC68-1–200, Flag-CCDC68-1–250, or Flag-CCDC68-1–300 and GFP-CDC20 were subjected to IP with an anti-GFP antibody and analyzed by immunoblotting with the indicated antibodies. (**I**) HEK293T cells coexpressing Flag-CCDC68-Full length (FL), Flag-CCDC68-1–300, Flag-CCDC68-1–290, Flag-CCDC68-1–280, Flag-CCDC68-1–270, or Flag-CCDC68-1–260 and GFP-CDC20 were subjected to IP with an anti-GFP antibody and analyzed by immunoblotting with the indicated antibodies. (**J**) HEK293T cells coexpressing Flag-CCDC68-1–300, Flag-CCDC68-Δ280–300, Flag-CCDC68-Δ280–290, or Flag-CCDC68-Δ290–300 and GFP-CDC20 were subjected to IP with an anti-GFP antibody and analyzed by immunoblotting with the indicated antibodies. (**K**) Schematic diagram of CCDC68-Full length (FL), truncated CCDC68 mutants, and CCDC68 deletion mutants. CDC20 binding ability: +, positive; -, negative. (**L**) Lysates of HEK293T cells coexpressing Flag-CCDC68-Full length (FL) or Flag-CCDC68-Δ280–300 and GFP-CDC20 were subjected to IP with an anti-GFP antibody, and the resulting samples were analyzed by immunoblotting with the indicated antibodies. (**M**) Schematic diagram of CDC20-WT and CDC20-IRAA. IRAA: I498A and R499A. CCDC68 binding ability: +, positive; -, negative. (**N**) HEK293T cells coexpressing GFP-CCDC68 and Flag-CDC20-WT or Flag-CDC20-IRAA were subjected to IP with an anti-GFP antibody and analyzed by immunoblotting with the indicated antibodies. (**O**) WT, *CCDC68*-KO, and *CCDC68*-KO cells stably expressing Flag-CCDC68-Full length (FL) or Flag-CCDC68-Δ200–220 were synchronized to prometaphase and subjected to immunoprecipitation with an anti-CDC20 antibody, and the samples were analyzed by immunoblotting with the indicated antibodies. All the data are presented as the means of the indicated biological replicates; error bars represent the means ± SEMs. Statistical analyses were performed using Student’s t test for (**C**) and (**F**). n.s., not significant, *****P* < 0.0001.

**Supplementary information, Fig. S6. CCDC68 limits APC/C-dependent ubiquitinated degradation by inhibiting MCC disassembly. Related to Fig. 4.**


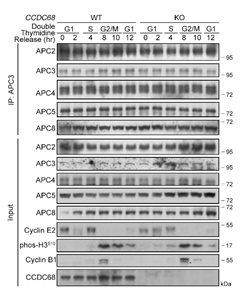


Wild-type (WT) and *CCDC68*-knockout (KO) HeLa cells were synchronized by double-thymidine block. The cells were then harvested, the lysates were subjected to immunoprecipitation with an anti-APC3 antibody, and the samples were analyzed by immunoblotting with the indicated antibodies.

**Supplementary information, Fig. S7. CCDC68 stabilizes the MCC by inhibiting the ubiquitination-dependent turnover of CDC20. Related to Fig. 5.**


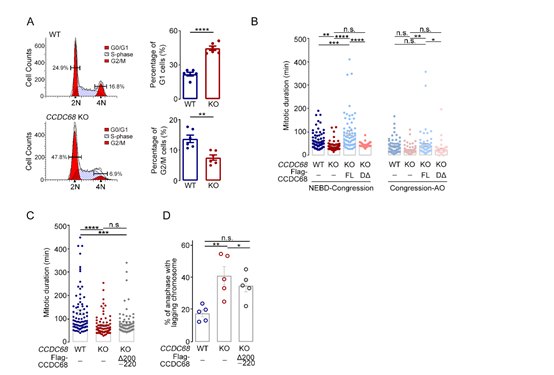


(**A**) Wild-type (WT) and *CCDC68*-knockout (KO) cells were subjected to flow cytometry analysis. Quantification of the percentage of cells in the G1 or G2/M phase. (**B**) Time from nuclear envelope breakdown (NEBD) to congression and time from congression to anaphase onset were quantitated from the time-lapse analysis shown in Figure **5G**. NEBD: nuclear envelope breakdown; AO: anaphase onset. (**C**) The time from NEBD to anaphase onset was quantified via time-lapse analysis of wild-type (WT), *CCDC68*-knockout (KO), and *CCDC68*-KO HeLa cells stably expressing Flag-CCDC68-Δ200–220. (**D**) Quantification of the percentage of cells exhibiting mitotic slippage in the time-lapse analysis of wild-type (WT), *CCDC68*-knockout (KO), and *CCDC68*-KO HeLa cells stably expressing Flag-CCDC68-Δ200–220. All the data are presented as the means of the indicated biological replicates; error bars represent the means ± SEMs. Statistical analyses were performed using Student’s t test for (**A**) and using one-way ANOVA for (**B**), (**C**), and (**D**). n.s., not significant. **P* < 0.05, ***P* < 0.01, ****P* < 0.001, *****P* < 0.0001

**Supplementary information, Fig. S8. Downregulation of CCDC68 induces aneuploidy. Related to Fig. 6.**


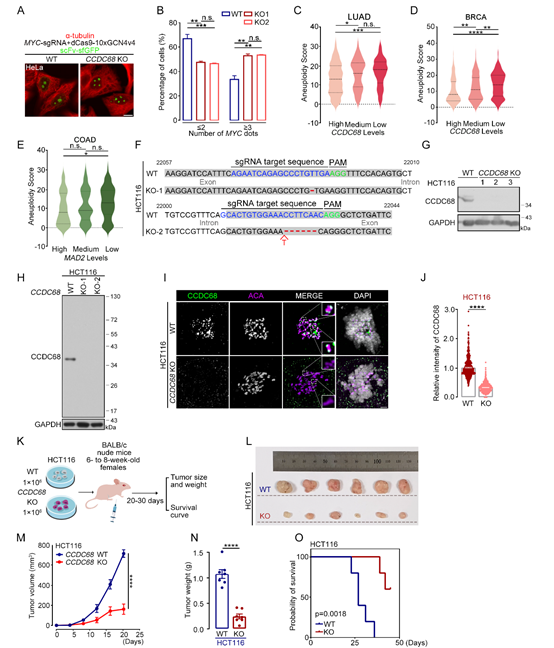


(**A**) Wild-type (WT) HeLa cells or *CCDC68*-knockout (KO) HeLa cells were transfected with *MYC*-sgRNA, dCas9-10×GCN4v4 and scFV-sfGFP for 24 h. Immunostaining for α-tubulin (red). Scale bar, 10 μm. (**B**) Quantification of the percentage of cells with the indicated *MYC* dots in (**A**). (**C–E**) Bioinformatics analysis of the association of the aneuploidy score with CCDC68 mRNA expression (**C** and **D**) and MAD2 mRNA expression (**E**) in patients with breast cancer, lung cancer or colon cancer. The mRNA expression data were downloaded from the TCGA database (https://www.cbioportal.org/). BRCA: Breast invasive carcinoma; LUAD: lung adenocarcinoma; COAD: colon adenocarcinoma. (**F**) Schematic of WT and *CCDC68*-KO HCT116 cell lines. The protospacer adjacent motif (PAM) sequence is highlighted in green, the sgRNA target sequence is highlighted in blue, and the mutated sequence is highlighted in red. The sgRNA deleted the exon‒intron splice site, which was accompanied by frameshift indels in the targeted region. (**G**) Immunoblotting of lysates from WT and *CCDC68*-KO HCT116 cells. GAPDH served as the loading control. (**H**) Immunoblotting of lysates from WT and *CCDC68*-KO HCT116 cells. GAPDH served as the loading control. (**I**) Immunostaining for CCDC68 (green) and ACA (purple) in WT or *CCDC68*-KO HCT116 cells. DNA was stained with DAPI (white). Scale bar, 2 μm. (**J**) Quantification of the fluorescence intensity of CCDC68 staining on kinetochores shown in (**I**). More than 100 kinetochores from 10 cells were analyzed. (**K**) Schematic diagram showing the experimental design for the xenograft mouse models. (**L**) Photographs of tumors from each group of mice (n = 6) at the end of the experiment. (**M**) Graph of tumor volume measured at different time points following the injection of WT and *CCDC68* KO HCT116 cells (n = 6). (**N** and **O**) WT and *CCDC68* KO HCT116 cells were injected into nude mice for evaluation of xenograft growth. The tumor weights (**N**) were measured, and a survival curve (**O**) was generated. n = 6 mice per group. All the data are presented as the means of the indicated biological replicates; error bars represent the means ± SEMs. Statistical analyses were performed using one-way ANOVA for (**B**–**E**) and using Student’s t test for (**J**) and (**M**–**O**). n.s., not significant. **P* < 0.05, ***P* < 0.01, ****P* < 0.001, *****P* < 0.0001
